# Covalent Positioning of Single DNA Molecules for Nanopatterning

**DOI:** 10.3390/nano11071725

**Published:** 2021-06-30

**Authors:** Eung-Sam Kim, Jung Sook Kim, Nishan Chakrabarty, Chul-Ho Yun

**Affiliations:** 1 Department of Biological Sciences, Research Center of Ecomimetics and Center for Next Generation Sensor Research and Development, Chonnam National University, Gwangju 61186, Korea; 2 Department of Chemistry, Division of Integrative Biosciences and Biotechnology, Pohang University of Science and Technology, Pohang 37673, Korea; chastejs@postech.ac.kr; 3 School of Biological Sciences and Biotechnology, Chonnam National University, Gwangju 61186, Korea; nishaniiuc@gmail.com (N.C.); chyun@jnu.ac.kr (C.-H.Y.)

**Keywords:** molecular writing, DNA ligation, covalent bond, atomic force microscopy, dendron-coated surfaces, DNA-based nanomachine

## Abstract

Bottom-up micropatterning or nanopatterning can be viewed as the localization of target molecules to the desired area of a surface. A majority of these processes rely on the physical adsorption of ink-like molecules to the paper-like surface, resulting in unstable immobilization of the target molecules owing to their noncovalent linkage to the surface. Herein, successive single nick-sealing facilitated the covalent immobilization of individual DNA molecules at defined positions on a dendron-coated silicon surface using atomic force microscopy. The covalently-patterned ssDNA was visualized when the streptavidin-coated gold nanoparticles bound to the biotinylated DNA. The successive covalent positioning of the target DNA under ambient conditions may facilitate the bottom-up construction of DNA-based durable nanostructures, nanorobots, or memory system.

## 1. Introduction

Historically, human beings have inscribed or written letters and symbols on solid surfaces using sharp tools or pens to record and share information. In terms of nanofabrication, the top-down method can be viewed as an approach where unnecessary parts are removed from the substrate to form nanostructures whereas the bottom-up method adds building blocks onto the substrate to construct nanostructures. Since the 20th century, various top-down approaches, such as UV-based photolithography [1], e-beam lithography [2], imprint lithography [3], and soft lithography [4], have been developed to implement high-density patterning on a small surface area. In 1959, Feynman envisioned writing 24 volumes of the Encyclopedia Britannica on the head of a pin [5], suggesting the potential of atomic or molecular bottom-up manipulation. At the molecular level, writing with a pen on a paper can be considered as the transfer of ‘ink’ molecules from its tip to the ‘paper’ surface.

Recently, the bottom-up approaches for atomic and molecular writing were demonstrated at the sub-nanometer and nanometer scales, respectively. Using a scanning tunneling microscope (STM), single Xe and Br atoms or Cu-TBP-porphyrin molecules adsorbed on a metal surface could be rearranged by exerting a finite force on them [6,7,8]. The STM facilitated the precise and reversible placement and removal of individual organic molecules on a graphite surface [9]. Atomic force microscopy was adopted in dip-pen nanolithography for transferring the ink molecules from the tip of the microscope to a surface when the tip was close to the surface. Furthermore, enzyme-assisted nanolithography via atomic force microscopy could locally position the product precipitate from the in-solution substrate [10,11]. The combination of DNA hybridization and atomic force microscopy (AFM) implemented the bottom-up assembly of DNA oligomers that were repeatedly picked up from a depot and deposited on a target area [12].

However, single atoms, molecules, or the molecular complex patterns created with these bottom-up methods adhered to the surface by physical bonds, and not covalent bonds. The covalent bond found in biological molecules is known to have a higher bond strength (at least ten times greater) than other chemical interactions such as hydrogen bonds, ionic bonds, Van der Waals forces, and hydrophobic interactions [13,14]. The weak and noncovalent immobilization of single molecules on the surface might lead to their undesired migration or detachment. In case of DNA robotics, the precise and stable localization of surface-bound DNA allowed the execution of defined tasks, such as autonomous movement [15], cargo transport [16], and DNA-template synthesis [17]. Additionally, most works with DNA data storage have relied on the phosphoramidite-based synthesis, which may bring errors due to the side reactions that limit the length of oligonucleotides [18]. The single step immobilization of oligonucleotides on the solid surface can be an alternative to the conventional method in DNA data storage. Therefore, the covalent positioning of single DNA strands is essential for constructing desired patterns with greater stability for single-molecule biophysics and nanobiodevices.

Our group has characterized the mechanical forces between probe biomolecules such as DNA, enzymes, antibodies, or signaling proteins, and their interacting partners in the solution using atomic force microscopy [19,20,21,22]. The surface modification with 27-acid or 9-acid dendron molecules was found to be critical in securing the lateral spacing (6.5 and 3.2 nm) of the probe and partner molecules immobilized on the AFM tip and the bottom surface, respectively [19,23]. The regular lateral spacing of the multiple surface-immobilized DNA strands allowed us to investigate the kinetics of on-chip DNA ligation. Furthermore, this modification significantly increased the probability of observing and mapping the desired single interactions between the AFM tip and the surface. We previously reported the in situ monitoring of single DNA nick-sealing by the enzymatic action of DNA ligase present in the reaction environment [24]. The work suggested the possibility of single-molecule patterning based on DNA ligation, which resulted in a covalent bond of patterned DNA molecules on the surface. In this study, the atomic force microscopy-based DNA ligation was conducted successively at selected positions on the dendron-coated bottom surface. The location of the ligated DNA strands was validated individually via force mapping around the DNA-ligated points. Lastly, the localization of all ligated DNA strands was visualized with gold nanoparticles which were bound to the ligated DNA through the interaction of streptavidin and biotin.

## 2. Materials and Methods

### 2.1. Materials

All chemicals and solvents were purchased from Sigma-Aldrich (St. Louis, MO, USA), unless otherwise specified. Ultra-pure water (18 × 10^6^ Ω∙cm) was supplied from a Milli-Q purification system (Millipore, Burlington, MA, USA). The polished prime Si wafer (crystalline orientation: <100>, n type dopant: phosphorus, resistivity: 1.5 to 2.1 Ω∙cm) was purchased from MEMC Electronic Materials Inc. (Seoul, Korea). The wafer was fragmented into rectangular (3 cm × 1 cm) substrates using a diamond scriber. AFM probes were obtained from NanoInk (DPN Probe: type B, Skokie, IL, USA). The silane coupling agent N-(3-(triethoxysilyl)propyl)-O-polyethyleneoxide urethane (TPU) was purchased from Gelest (Morrisville, PA, USA). The two types of dendron molecules, 9-anthrylmethyl-3-({[tris({[(1-{tris[(2-{[(tris{[2-carboxyethoxy]methyl}ethyl)amino]carbonyl}ethoxy)-methyl]methyl}amino)carbonyl]-2-ethoxy}methyl)methyl]amino}-carbonyl)propylcarbamate and 9-anthrylmethyl *N*-({[tris({2-[({tris-[(2-carboxyethoxy)methyl]methyl}amino)carbonyl]ethoxy}methyl)-methyl]amino}carbonyl)propylcarbamate, were custom-synthesized at Panagene (Daejeon, Korea) and referred to as 27-acid and 9-acid dendrons, respectively. The single-stranded nucleotides were synthesized, chemically modified at the termini by adding phosphate (PO_4_), hydroxyl (OH), or amine (NH_2_) groups, or biotin, and were purified at Bionics (Seoul, Korea). The oligonucleotide sequences used for the experiment were as follows: 24mer (5′: PO_4_, 3′: NH_2_), 5′-PO_4_-CCGCTCGTGCTCATCATAGTAACC-NH_2_-3′; 47mer (5′: biotin), 5′-biotin-CCCTGAGTCTACCTAATATGACTACTAATCGTGCTGATGCCTCAGTG-3′; c71mer (3′: NH_2_), 5′-GGTTACTATGATGAGCACGAGCGGCACTGAGGCATCAGCACGATTAGTAGTCATATTAGGTAGACTCAGGG-NH_2_-3′. The sequences of the three single-stranded DNAs (24mer, 47mer, and c71mer DNAs) were designed to minimize the formation of secondary structures in the ligation solution. T4 DNA ligase (400 U/μL) and adenosine triphosphate (ATP, 100 mM in stock, purity of >96% by HPLC) were obtained from Solgent (Daejeon, Korea) and Fermentas (GlenBurnie, MD, USA), respectively. Streptavidin-coated gold nanoparticles (40 nm in diameter) and the dilution buffer were purchased from KPL (Gaithersburg, MD, USA).

### 2.2. Dendron Modification on AFM Tips and Silicon Substrates

The AFM probes (tip radius ~15 nm; nominal spring constant: 16 pN/nm) were modified with the 27-acid dendron molecule, whereas the silicon substrates were modified with the 9-acid dendron molecules, as previously described [19]. Briefly, the oxidized AFM probe and the silicon substrate were coated with TPU to generate hydroxyl groups on the surface. The subsequent esterification reaction linked the carboxyl groups present at the periphery of dendron molecules to the hydroxyl groups present on the solid surface. The protecting group (that is, 9-9-anthrylmethoxycarbonyl group) present at the apex of the immobilized dendron was removed chemically to expose the amine groups.

### 2.3. Immobilization of ssDNA on AFM Tips and Silicon Substrates

The deprotected surface of the immobilized dendron was immersed in an acetonitrile solution containing di(*N*-succinimidyl)carbonate (DSC, 25 mM) and diisopropylethylamine (1 mM) for 3 h in a nitrogen atmosphere to tether the *N*-hydroxysuccinimide (NHS) group to the terminal. The AFM tip and silicon substrate were subjected to stirring in dimethlylformamide for 1 h, rinsed with methanol, and dried under vacuum conditions (ca. 50 mTorr). The NHS-functionalized AFM tip was incubated in a spotting buffer (25 mM sodium bicarbonate, 5 mM MgCl_2_, and 10% (*v*/*v*) dimethyl sulfoxide, pH 8.5) containing 3′-NH_2_-modified c71mer DNA (20 μM) at 25 °C for 12 h, washed with a washing buffer (300 mM NaCl, 20 mM Na_2_HPO_4_, 2 mM ethylenediaminetetracetic acid, and 7 mM sodium dodecyl sulfate (SDS)), rinsed in deionized (DI) water, and dried under vacuum conditions. The 3′-NH_2_-modified 24mer DNA was dissolved in the spotting buffer, and the concentration was adjusted to 15 μM for immobilization on the NHS-functionalized silicon substrate. The ssDNA was then spotted on a glass substrate using a pin-based contact microarrayer (QArray mini; Genetix, New Milton, UK). The metallic pins were moved in proximity to a printing substrate whereby direct contact between the tips of pins and the surface resulted in the transfer of a small volume of the ssDNA solution on the substrate. The substrate was incubated in an 80% humidity chamber for 12 h and then washed with saline sodium citrate (SSC) buffer (150 mM NaCl, 15 mM sodium citrate, 0.2% SDS, pH: 7.0 at 25 °C) to remove unbound DNA. The substrate was rinsed with DI water and dried by centrifugation for 3 min at 200× *g* and 25 °C.

### 2.4. Successive Single DNA Nick-Sealing

**Figure 1:**
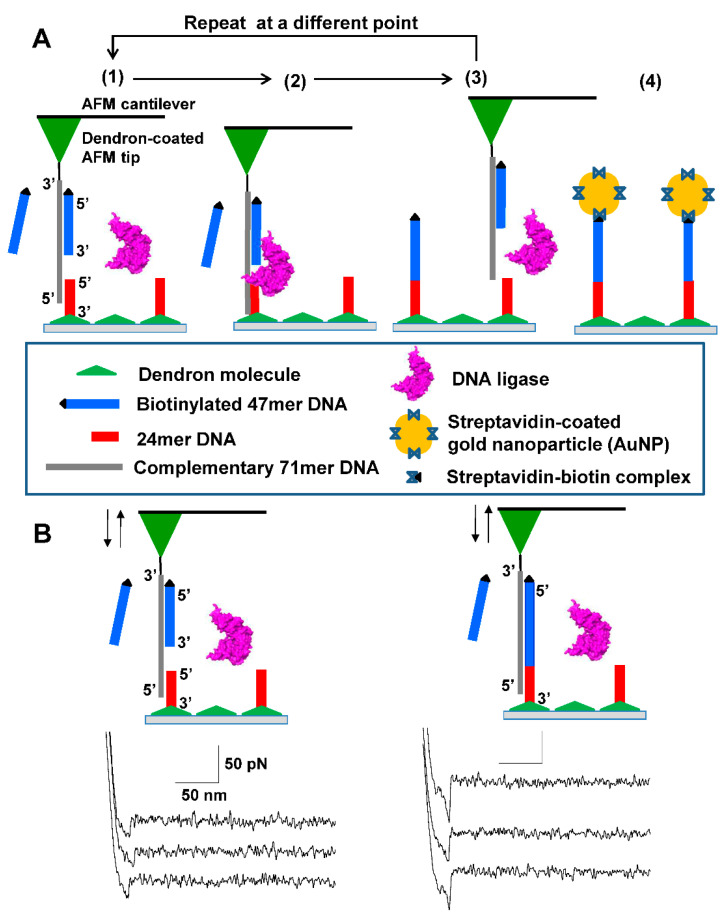
**Successive DNA ligation for covalently positioning single DNA molecules.** (**A**) Scheme of four-step process: (**Step 1**) A free 47mer DNA (in blue) modified with a biotin residue (represented by the black triangle) linked at its 5′ end is hybridized to the complementary 71mer (c71mer) DNA (in gray) immobilized on the tip of the AFM cantilever in the DNA ligation solution; (**Step 2**) The AFM tip is made to approach a 24mer DNA immobilized on the dendron-coated surface to introduce a single nick between the 5′ end (that is, a phosphate group) of the 24mer DNA and the 3′ end (that is, a hydroxyl group) of the 47mer DNA. The nick can be sealed by the DNA ligase, which forms a new phosphodiester bond in the nick; (**Step 3**) After a pause of 30 s, the AFM tip is pulled upward to allow the hybridization of the c71mer DNA with a new free 47mer DNA. The direct immobilization of a single 47mer DNA is performed repeatedly at different points for DNA patterning; (**Step 4**) Prior to visualizing the nanoscale DNA patterns, the streptavidin-coated gold nanoparticles are employed to label the ligated DNA, taking advantage of the high affinity between streptavidin and biotin. The wide triangle (in green) on the bottom surface represents the 27-acid dendron molecules coated on the silicon substrate. (**B**) Representative three single-peaked force-distance curves for the unbinding events between the 24mer DNA on the 9-acid dendron-coated bottom surface and hybridized c71mer/47mer DNA on the 27-acid dendron-coated AFM tip (left) and those between the ligated 71mer DNA and the c71mer DNA (right).

The formation of a single DNA nick, the enzymatic DNA ligation, and the in situ detection of single nick-sealing was performed using the NanoWizard AFM (JPK, Berlin, Germany). According to the AFM manufacturer, the lateral resolution is less than 2 nm while the vertical (i.e., z-directional) directional is less than 0.5 nm. The details of the AFM set-up, position control strategy for the AFM tip, and classification of force-displacement curves were described in our previous study [24]. Briefly, the AFM tip with a c71mer DNA was made to approach the 24mer DNA spot on the silicon substrate (Figure 1A). Two hundred microliters of the 1× ligation solution (30 mM Tris-HCl, 10 mM MgCl_2_, 10 mM 1,4-dithiothreitol, 1 mM ATP, pH 7.8) containing T4 DNA ligase (1 µM) and 47mer DNA (2 µM) was slowly injected into the gap between the AFM tip and the substrate. The force measurement over the 24mer spot was repeated with the approach/retraction cycle of 1.8 s until the unbinding force (ca. 25 picoNewton (pN)) for the 24 bp was measured. The z-length of the AFM tip was reduced to 20 nm at the point where the unbinding force was detected. The AFM tip was paused for 30 s and was then retracted to the initial z-length of 450 nm until the unbinding force for 71 bp measured as ca. 65 pN. When the specific unbinding force was observed to increase, the AFM tip was incubated in the ligation solution for 2 min to facilitate the hybridization of the 47mer DNA with the c71mer DNA. The AFM tip with the 47mer/c71mer DNA was shifted to another point on the 24mer DNA spot for the next single nick-sealing event.

### 2.5. Force Mapping via Atomic Force Microscopy

The AFM tip with the c71mer DNA was programmed to scan the area surrounding the ligated 71mer DNA. The scan area was divided into 20 × 20 or 10 × 10 sections (5 nm × 5 nm per section). In each section, five force-distance curves were successively plotted. The unbinding force was averaged for three or more curves with specific unbinding profiles.

### 2.6. Visualization of Points for the Ligated 71mer DNA

The streptavidin-coated AuNPs were diluted 50-fold with 1× phosphate-buffered saline (pH: 7.4 at 25 °C) and placed on the 24mer DNA spot on the silicon substrate for 30 min at 25 °C. After the incubation, the silicon substrate was sequentially rinsed with SSC buffer and DI water to remove the unbound AuNPs. Finally, the substrate was dried under vacuum conditions. The surface of the silicon substrate was imaged using a field-emission scanning electron microscope (FE-SEM, S-4800, Hitachi, Tokyo, Japan). When we ligated single DNAs using AFM, the microscope equipped with a motorized stage displayed the planar coordination of the AFM tip on the silicon substrate at submicron precision. The mean AFM coordination was applied to the SEM imaging. Due to the measurement error in the planar position of the two independent equipments, the repeat of zoom-in and zoom-out around the mean point to pinout the nanopatterned area.

## 3. Results and Discussion

We performed successive single nick-sealing in the DNA duplex using an AFM under ambient conditions. To immobilize the 24mer and c71mer DNAs on the silicon substrate and AFM tip surfaces, we coated the surfaces with the 9-acid and 27-acid dendrons, respectively. The force-distance curve obtained from repeated force picking (that is, the approach of the AFM tip toward the silicon substrate and its subsequent vertical retraction) at a point determined the hybridization of the 24mer and c71mer DNAs in the ligation solution containing the biotinylated 47mer DNA and the DNA ligase. There was a trade-off between securing the single nick-sealing events and promoting the binding probability of c71mer DNA to 24mer DNA. When both the AFM tip and bottom surface were modified with 27-acid dendron molecules for a larger lateral spacing, we could effectively avoid multiple nicking events. However, the probability for the binding events was significantly decreased, which required us to spend much more time in ligating DNAs at a point. On the other hand, when both the AFM tip and bottom surface were coated with 9-acid dendron molecules, we could obtain a high probability of the binding events which adversely provoked multiple nick-sealing events under AFM tip. Since multiple nick-sealing events prevented us from making single molecular nanopatterns, we finally decided to modify the AFM tip and bottom surface with 27-acid and 9-acid dendron molecules, respectively.

The three DNA strands hybridized to form a single DNA duplex with a single nick between the 3′ end of the 47mer DNA and the 5′ end of the 24mer DNA (steps (1) and (2) in Figure 1A). This nick would be repaired by DNA ligase during the pause time of 30 s to form a 71 bp DNA duplex. Based on the optimized conditions for single nick-sealing events previously describes by our group [24], a transition of unbinding forces from ca. 25 to 65 pN was observed when the AFM tip was retracted after the pause time as shown in Figure 1B. This transition in force indicates the covalent nick-sealing at the single-molecule level. The subsequent incubation of the AFM tip in the ligation solution for 2 min induced the hybridization of another 47mer DNA (freely diffusing in the solution) to the c71mer DNA on the AFM tip (step (3) in Figure 1A). The AFM tip with the 47mer/c71mer DNA was shifted to a different position on the silicon substrate according to a DNA positioning (or DNA patterning) map for the next single nick-sealing event. The average distance between the two neighboring positions was set to 250 nm. We aimed to create an ‘N’-shaped pattern on the silicon substrate using the covalently immobilized 71mer DNAs through repeated nick-sealing at each desired position. In the final step, the streptavidin-coated AuNPs were used to visualize the position of the covalently immobilized 71mer DNA with the terminal biotin moiety (step (4) in Figure 1A).

We classified the force-distance curves into five DNA/DNA unbinding events in terms of the number of specific peaks: non-specific unbinding, no binding, single peaked unbinding, double peaked unbinding, and multiple peaked unbinding. The multiple peaked binding events had three or more peaks in a given force-distance curve. Prior to the DNA ligation, we could see ca. 77% for single-peaked unbinding force of 24.2 ± 3.7 pN, 4% for double peaked unbinding force of 25.1 ± 4.2 pN, and 9% for no binding out of more than 500 force picking trials at least five different points. After the DNA ligation, the similar distribution of unbinding events were obtained (i.e., 83% for single-peaked unbinding, 8% for double-peaked unbinding, and 9% for no binding) whereas the unbinding force for the single- and double-peaked unbinding events increased to 63 ± 15.6 pN and 65 ± 13.2 pN, respectively. The dendron modification could bring us a high probability of single peaked unbinding events even though the 47mer DNA was dispersed in the ligation solution.

Our preliminary test for on-chip DNA ligation [25] and the subsequent incubation with either Cy5-labeled streptavidin or streptavidin-coated AuNPs confirmed the specific interaction between streptavidin and the terminal biotin moiety of the ligated DNA on the dendron-coated silicon substrate. The spot for the ligation of the biotinylated 47mer DNA emitted a Cy5 fluorescence signal, while the spot with non-biotinylated 47mer DNA emitted no detectable fluorescence (Figure S1). The interaction of the streptavidin-coated AuNPs with biotinylated DNA facilitated the visualization of the complex via AFM topography (Figure S2), confirming that their affinity was sufficiently high and specific [26] to withstand the washing step. The biotinylated DNA spot with a diameter of ca. 100 µm contained overwhelming numbers of AuNPs compared to the non-biotinylated DNA spot. Individual AuNPs could be topographically profiled in the biotinylated DNA spot.

**Figure 2:**
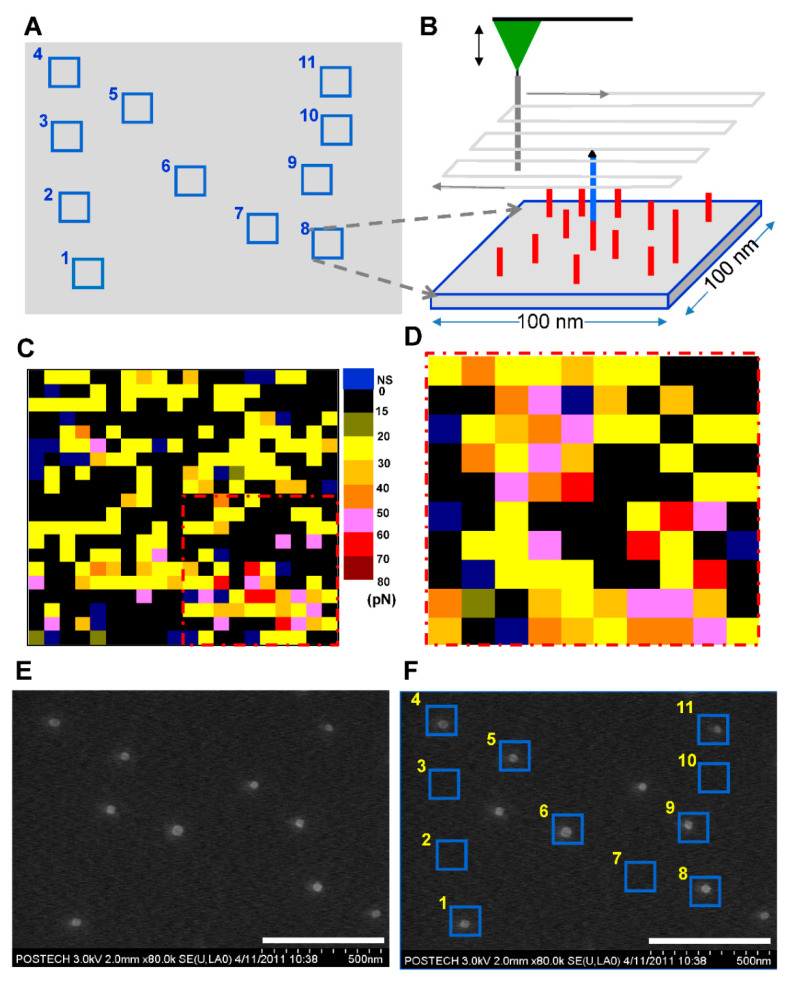
**Visualization of the positions of the 71mer DNA.** (**A**) DNA positioning map: successive single DNA nick-sealing was performed at different positions in the 1.5 µm × 1.1 µm area to create an N-shaped pattern. The center of each square of the positioning map was made to coincide with the point at which the single DNA nick-sealing was validated. (**B**) Scheme for AFM force mapping: an AFM tip with the c71mer DNA scanned the surface of a square depicted in (**A**) to confirm the presence of the ligated 71mer DNA with the 5′-biotin. (**C**) Representative force map: the unbinding force (from 0 to 80 pN) recorded in each section of a square was indicated by the color scale. NS: non-specific unbinding force. (**D**) Force map obtained from the 2nd round of force mapping of the region dashed in red in (**C**). (**E**) SEM image of the streptavidin-coated AuNPs on the silicon substrate. (**F**) Overlap of the SEM image with the DNA positioning map. Scale bars: 500 nm in (**E**,**F**).

Based on these preliminary results, we attempted to link the biotinylated 47mer DNA to the 24mer DNA individually at 11 different points by adopting the atomic force microscopy-based nick-sealing approach to construct a nanoscale ‘N’-shaped pattern composed of DNA-AuNPs. When the force transition was observed at a point upon the retraction of the AFM tip, the planar coordinate of the point where the AFM tip was positioned during the pause was recorded to construct the position map (Figure 2A). The force mapping was performed to validate the presence of the ligated 71mer DNA at the force transition point on the silicon substrate (Figure 2B). We applied the 30-s-long pause during each DNA ligation at a point. When free 47mer DNA probes were dispersed in the ligation solution, the nick-sealing probability was decreased to ca. 30% compared to the single nick-sealing probability of 60% [24] in the absence of the 47mer DNA in the ligation solution. It is not clear why the yield decreased from 60% to 30% or less in the successive ligation under the pause of 30 s. The free 47mer DNA probe might interfere the hybridization or enzymatic reaction in the solution.

An AFM tip with the c71mer DNA scanned over the region surrounding (area: 100 nm × 100 nm) each force transition point. Five force-distance curves were collected on each section of 5 nm × 5 nm in area. The unbinding force was extracted when the curve was determined to be valid to show specific single unbinding event. When the non-specific unbinding curve was discarded, the mean of the unbinding forces in each section ranged from 0 to 80 pN, as shown in the color-coded force map (Figure 2C). For region No. 8, the unbinding force (that is, 60 to 70 pN) corresponding to the 71 bp DNA duplex was detected in five sections in the lower right section, while the other sections presented lower unbinding forces. The 2nd-round force scan over the lower right corner created a force map that depicted the clustered sections with the unbinding force of 50 to 70 pN (Figure 2D). The difference between the two force maps for the identical area might be attributed to the Brownian motion of the 71mer DNA and the thermal drift of the AFM tip. Relatively larger unbinding forces were measured in the central sections, whereas smaller unbinding forces were detected in the sections surrounding the circular cluster with its diameter of ca. 25 nm, which was consistent with the finding of our previous report [23] on force mapping for surface-immobilized DNA. Because of the thermal drift of the 71mer DNA in the solution, the position of the 71bp DNA unbinding event was not fixed at a single section as shown in the dashed region in Figure 2C,D. Although they seemed to be noisy, the planar range of the pink- or red-colored sections coincided with the hydrodynamic radius of the 71mer DNA at room temperature.

**Figure 3:**
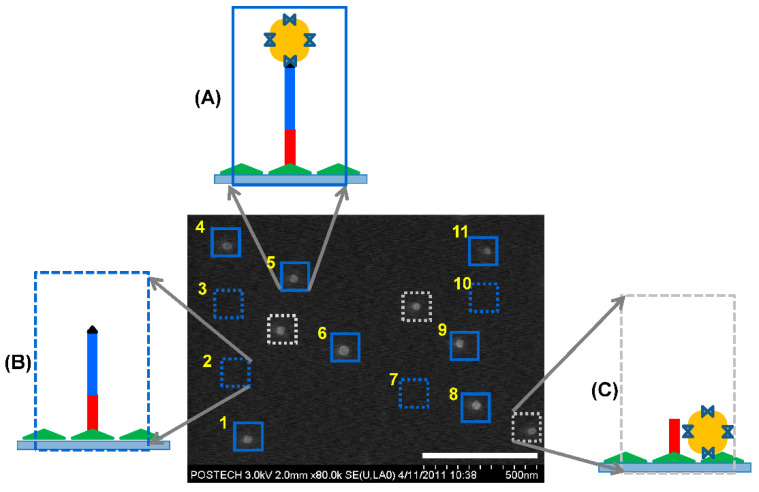
**Evaluation of the DNA-AuNP complexes.** (**A**) A single streptavidin-coated AuNP was imaged in the area surrounding a point where a single 71mer DNA with a terminal biotin moiety was covalently immobilized. The position of the DNA-AuNP complexes is indicated by the blue-lined square. (**B**) No AuNP was detected at a point where a single 71mer DNA was positioned. The position of a single DNA without AuNP is indicated by the blue-dotted square. (**C**) A AuNP was detected outside the DNA positioning map. The position of the AuNPs non-specifically bound to the silicon substrate is indicated by the gray-dotted square.

The interaction between the streptavidin-coated AuNP and the biotinylated 71mer DNA on the silicon substrate allowed us to visualize the position of the covalently immobilized DNA using scanning electron microscopy (SEM) (Figure 2E). We avoided AFM topography because the physical interaction between the AFM tip and the AuNPs could displace the AuNPs on the surface. When the position map of the 71mer DNA was merged with the SEM image (Figure 2F), the single AuNPs were localized at 7 sites out of 11. Four sites, including the regions No. 2, 3, 7, and 10, did not accommodate AuNPs. The surface-immobilized 71mer ssDNA present in the four sites was predicted to form secondary or worm-like chain structures at room temperature (Figure S3) with a minimum free energy [27]. If it were stretched to its full length of 49 nm (length per base of ssDNA: 0.676 nm, [28]), the AuNP bound to the 71mer DNA would have been located outside the site boundary. Three AuNPs were detected in the background of the N-shaped pattern. The overlap between the AuNPs and the DNA position map confirmed the presence of a single 71mer DNA at the force transition point (Figure 3A). The DNA region lacking AuNPs might represent the sites where the AuNPs were removed during the washing step, or those where there was no interaction between the biotinylated DNA and the streptavidin-coated AuNPs during the AuNP incubation step (Figure 3B). AuNPs detected outside the 71mer DNA region might have adhered by non-specific adsorption to the silicon substrate (Figure 3C). The AFM tip was laterally translated around 230 nm away from the existing points to minimize physical damages to the 71mer ligated DNA. It is expected that a more dense nanopattern can be constructed if the lateral translation of the AFM tip is reduced to ca. 60 nm when considering the hydrodynamic radius of the 71mer ligated DNA.

We attempted to construct an AuNP-47mer DNA conjugate that might be used as an ink-like molecule for the single-step nanopattening. It was constructed by the incubation of thiol-modified ssDNA with AuNPs and the subsequent gel separation of the 1:1 conjugate (that is, a single thiol-modified 47mer DNA bound to a single AuNP), based on our previous protocol [29]. Unfortunately, the force transition at the nick-sealing point was rarely observed when we employed the conjugate instead of the 47mer DNA. The low yield of the equimolar conjugate might drastically inhibit the hybridization between the conjugate and the c71mer DNA in the ligation solution. Additionally, the non-specific adsorption of AuNPs to the AFM tip might prevent the hybridization between the 24mer and c71mer DNAs.

## 4. Conclusions

The covalent positioning of single DNA molecules at defined positions for nanoscale patterning or nanowriting was demonstrated using the successive single DNA nick-sealing approach combining atomic force microscopy and dendron-based surface modification. The position of the immobilized DNA with the terminal biotin moiety could be confirmed by force mapping and it could be visualized with the streptavidin-coated AuNPs. The direct positioning of single DNA molecules on the solid surface through covalent bonding can be adopted in the bottom-up construction of DNA-based nanomachines or nanocircuits such as DNA tracks of DNA biped walkers and molecular digital data storage, substantially enhancing the reproducibility of their structures and functions.

## Data Availability

The data presented in this study are available on request from the corresponding author.

## Supplementary Materials

The following are available online at https://www.mdpi.com/article/10.3390/nano11071725/s1. On-chip DNA ligation; Fluorescence scanning and signal quantification; AFM topography; Figure S1: Confirmation of enzymatic DNA ligation via on-chip DNA ligation; Figure S2: AFM topographic images of the AuNP-DNA complexes; Figure S3: Prediction of the secondary structure of 71mer DNA at 25 °C.
