# Supplementary material for: Covalent Positioning of Single DNA Molecules for Nanopatterning
Source: Nanomaterials (Basel). 2021 Jun 30;11(7):1725. doi: 10.3390/nano11071725 (PMC8307146; doi:10.3390/nano11071725)
Supplement: Supplementary file 1 [file nanomaterials-11-01725-s001.zip › nanomaterials-1260293-SI.pdf]

nanomaterials

Supplementary Information

Covalent Positioning of Single DNA Molecules for Nanopatterning

Eung-Sam Kim [1,*], Jung Sook Kim [2], Chakrabarty Nishan [3] and Chul-Ho Yun [3]

1 Department of Biological Sciences, Research Center of Ecomimetics and Center for Next Generation Sensor Research and Development, Chonnam National University, Gwangju 61186, Korea
2 Department of Chemistry, Division of Integrative Biosciences and Biotechnology, Pohang University of Science and Technology, Pohang 37673, Korea; chastejs@postech.ac.kr
3 School of Biological Sciences and Biotechnology, Chonnam National University, Gwangju 61186, Korea; nishaniiuc@gmail.com (C.N.); chyun@jnu.ac.kr (C.-H.Y.)
* Correspondence: eungsam.kim@chonnam.ac.kr; Tel.: +82-62-530-3416; Fax: +82-62-530-3409

On-chip DNA ligation

The 9-acid dendron-coated glass slides (76 mm × 25 mm × 1 mm) were obtained from NSB POSTECH (Seoul, Korea). Lyophilized Cy5-labeled streptavidin (KPL, Gaithersburg, USA) was rehydrated according to the manufacturer's instruction (final concentration: 1.0 mg/mL, stored at 4 ºC) and diluted with PBS prior to use. The NHS functionalization, immobilization of the 24mer DNA, and hybridization of the 47mer/c71mer was performed following the protocol described in previous reports.1,2 Briefly, the 9-acid dendron-coated glass slide with the amine groups underwent NHS functionalization followed by the covalent linkage of the 3′-NH2-24mer DNA through pin-contact spotting (5-by-5 spots for biotinylated 47mer DNA and 5-by-5 spots for non-biotinylated 47mer DNA, as depicted in Fig. S1(B). The subsequent incubation of the 47mer and c71mer DNA solutions in the 24mer DNA spot led to the introduction of a single nick in the hybridized DNA. The T4 DNA ligase in the ligation buffer (30 mM Tris-HCl, 10 mM MgCl2, 10 mM 1,4-dithiothreitol, 1 mM ATP, pH 7.8 at 25 ºC) was used to seal the nick between the 24mer and 47mer DNAs. The glass slide was washed with saline sodium citrate (SSC) buffer and rinsed with deionized (DI) water before it was dried under vacuum conditions.

Fluorescence scanning and signal quantification

The Cy5-labeled streptavidin stock was diluted to 5 µg/mL (that is, 1:200 dilution) with 1X PBS. The diluted solution was allowed to incubate in the spotting area of the glass slide for 30 min at 25 ºC to induce the affinity interaction between the Cy5-labeled streptavidin and the biotinylated 47mer DNA immobilized on the glass slide. The glass slide was washed with SSC buffer and rinsed with DI water before it was dried under vacuum conditions. Fluorescent signals from the DNA-ligated spots on the glass surface were detected using a confocal laser scanner (ScanArray Lite, GSI Lumonics) with a Cy5 channel (excitation at 633 nm, emission at 670 nm). The signal was detected using specific scanning parameters (laser power gain: 80%, PMT gain: 80%, scanning resolution: 5 µm, and focal depth: 2,000 µm). The quantification of Cy5 fluorescence was conducted using a microarray analysis program (ImaGene, BioDiscovery) with background subtraction and presented as a scaled pseudo-color display.

AFM topography

The atomic force microscope (AFM) topography was imaged using an AFM (Dimension 3100, Digital In-struments) equipped with an AFM probe (NCH type, NanoWorld, Switzerland). The spring constant and reso-nance frequency of the cantilever were 42 N/m

and 320 kHz, respectively. The radius of curvature of the tip was less than 8 nm. Topographic images of 100 µm × 100 µm (512 × 512 pixels) were obtained in air under the tapping mode at 25 ºC and visualized using a viewer program (NanoScopever. 7.0, Veeco). The scanning speed was set to 1 Hz for a single line.

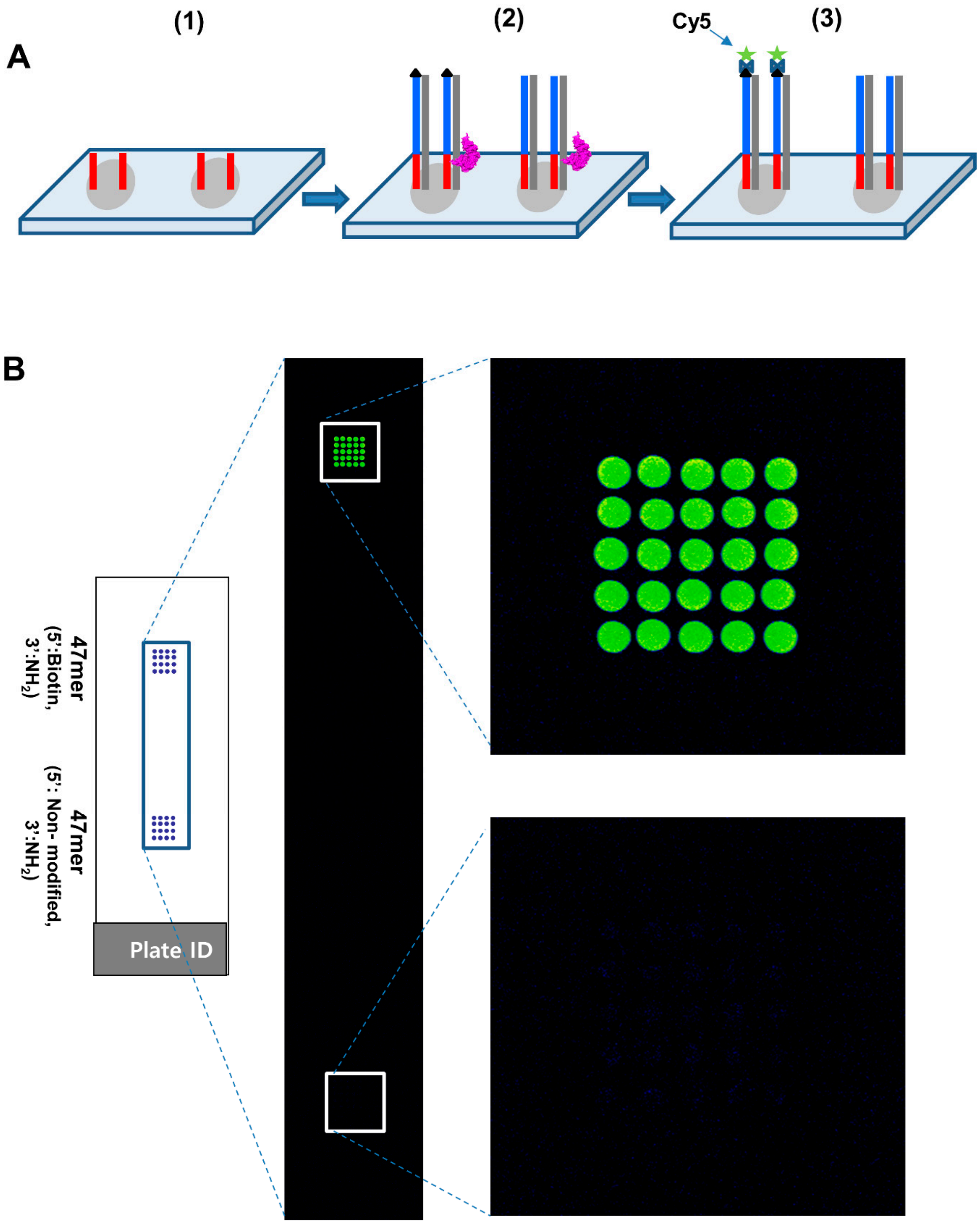

Figure S1. Confirmation of enzymatic DNA ligation via on-chip DNA ligation. (A) Overall procedure of on-chip DNA ligation followed by florescence detection. The 24mer DNA was immobilized on the dendron-coated surface using the spotting machine (1). The 47mer and c71mer DNAs were incubated in the spot to form the 71-bp complex comprising the three DNA strands using DNA ligase (2). Two types of c47mer DNA were recruited: biotinylated or non-biotinylated. The Cy5-labeled streptavidin in the dilution buffer was incubated and then washed with the wash solution to remove the free streptavidin (3). (B) Fluorescence images of the microarrayed spots. The 5-by-5 spots were arrayed in the solid substrate

(left). The upper and lower spots incubated with the DNA hybridization solution containing the biotinylated or non-biotinylated 47mer DNAs, respectively. After the hybridization, the Cy5 fluorescence signal was detected using a micro-array scanner (middle) and each spot region was enlarged (right). The spots had an average diameter of ca. 90 μm.

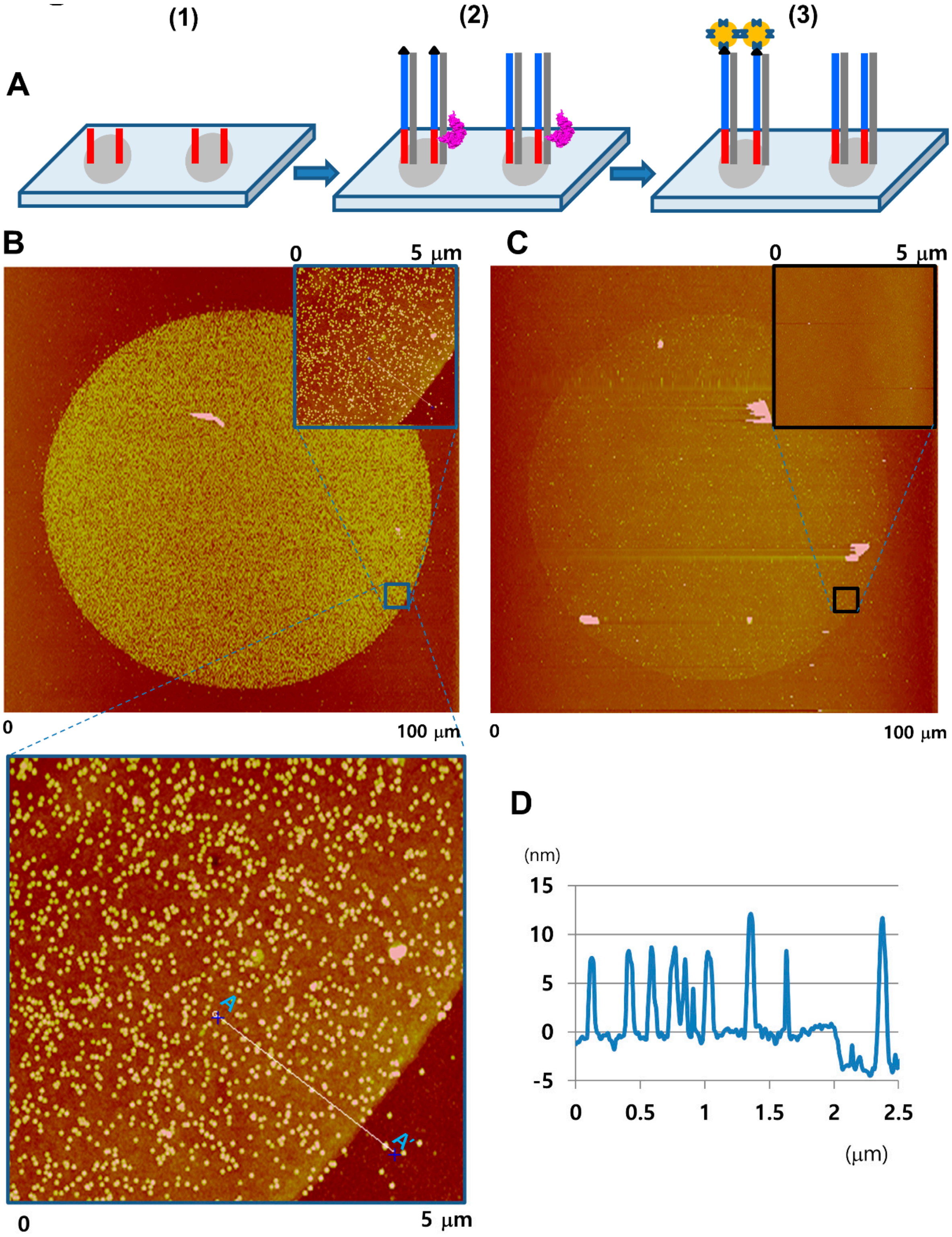

Figure S2. AFM topographic images of the AuNP-DNA complexes. (A) Overall procedure of on-chip DNA ligation followed by AFM topography. The 24mer DNA was immobilized on the dendron-coated surface using the spotting machine (1). The 47mer and c71mer DNAs were incubated in the spot to form the 71-bp complex comprising the three DNA strands using DNA ligase (2). Two types of 47mer DNA were recruited: biotinylated or non-biotinylated. The streptavidin-coated AuNPs in the dilution buffer were incubated and then washed with the wash solution to remove the free AuNPs (3). (B) The AFM image of a spot with biotinylated 47mer DNA and an enlarged image (inset, top right). (C) The AFM image of a

spot with non-modified 47mer DNA and an enlarged image (inset, top left). (D) A height profile along a line from A to A′ depicted in the enlarged image of the AuNP-DNA complex spot. .

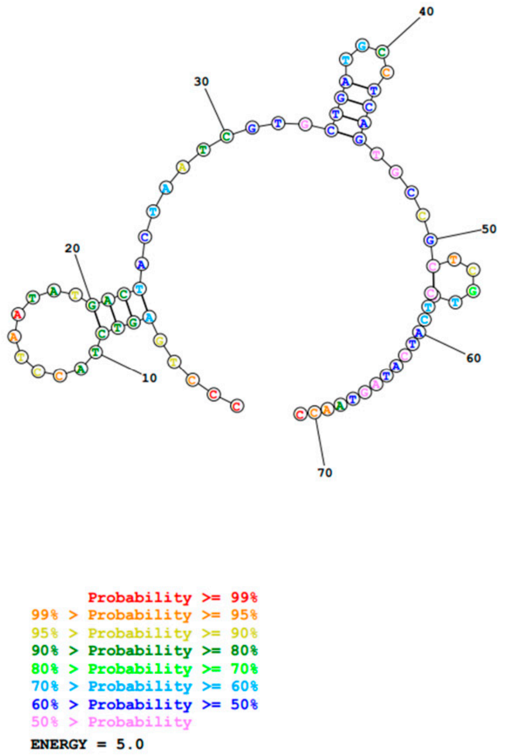

Figure S3. Prediction of the secondary structure of 71mer DNA at 25 ºC. The intrastrand hairpin structure was predicted using a web-based calculator named RNA structure (available at https://rna.urmc.rochester.edu/RNAstructureWeb/Servers/Predict1/Predict1.html).

Supplementary References

1. E. S. Kim: B. J. Hong, C. W. Park, Y. Kim, J. W. Park and K. Y. Choi, *Biosens Bioelectron*, 2011, **26**, 2566-2573.
2. E. S. Kim, N. Lee, J. W. Park and K. Y. Choi, *Nanotechnology*, 2013, **24**, 405703.
